# Factors associated with health-related quality of life in kidney transplant recipients in Korea

**DOI:** 10.1371/journal.pone.0247934

**Published:** 2021-03-11

**Authors:** Younghui Hwang, Misook Kim, Kyoungok Min

**Affiliations:** 1 Department of Nursing, College of Medicine, University of Ulsan, Ulsan, Korea; 2 Department of Mental Health Nursing, Seoul National University, Seoul, Korea; 3 Transplant Center, Department of Nursing, Seoul National University Hospital, Seoul, South Korea; Imperial College Healthcare NHS Trust, UNITED KINGDOM

## Abstract

Health-related quality of life (HRQOL) of kidney transplant recipients is an outcome evaluation after kidney transplants. Therefore, we investigated the associations among perceived health status, social support, self-determination, post-traumatic growth, and kidney transplant recipients’ HRQOL. This study involved a descriptive, self-report survey of 163 kidney transplant recipients visiting an outpatient solid organ transplant center in South Korea. Participants’ general and transplant characteristics, perceived health status, post-traumatic growth, social support, self-determination, and HRQOL were collected. Data were statistically analyzed using the software SPSS version 25.0. HRQOL showed statistically significant positive correlation with perceived health status (r = .56, p < .001), post-traumatic growth (r = .18, p = .022), social support (r = .25, p = .002), and self-determination (r = .36, p < .001). The factors affecting HRQOL were perceived health status (β = 0.47, p < 0.001), post-transplant occupation (β = 0.17, p = 0.009), and income source (β = -0.13, p = 0.046). The explanatory power of these variables was 34.8% (F = 28.81, p < 0.001). In the subdomains of HRQOL, the factors influencing HRQOL of mental component summary were perceived health status (β = 0.45, p < 0.001), self-determination (β = 0.27, p < 0.001), and education level (β = 0.18, p = 0.006). The explanatory power of these variables was 34.8% (F = 28.81, p < 0.001). To promote the HRQOL of kidney transplant recipients, an institutional system to assist kidney transplant recipients in returning to work needs to be developed. Additionally, creating an environment that allows kidney transplant recipients to act with self-determination, and developing intervention programs that can enhance self-determination will contribute to enhancing the HRQOL of kidney transplant recipients.

## Introduction

### Study significance

Kidney transplantation is a treatment that improves the health, health-related quality of life (HRQOL), and survival rate of patients with end-stage renal disease [1, 2]. The number of patients with end-stage renal disease awaiting a kidney transplant is growing, and the number of kidney transplants in Korea has increased from 1,289 in 2010 to 2,108 in 2018 [3].

The HRQOL is an outcome assessment undertaken after kidney transplants [4]. Therefore, efforts are being made to identify factors associated with the HRQOL of kidney transplant recipients [4–6], which includes the following: monthly income; religion [5]; perceived health status; post-transplant stress; social support [4]; and experienced symptoms such as fatigue, physical weakness, and reduced vision [7].

Kidney transplant recipients have to take immunosuppressants all their lives to prevent graft rejection [8]. However, this medication may cause them to be more susceptible to infections, fractures, and developing diseases such as diabetes and malignant tumors [9]. Therefore, they have to personally be responsible for their own health all their lives [10]. In this context, self-determination is an important factor in post-transplant self-management, which includes consistently taking immunosuppressants and managing one’s own health [11]. The self-determination theory argues that if the cause of a behavior is self-determined, it can induce intrinsic motives, making it easier to maintain the behavior [12]. In other words, if kidney transplant recipients undertake self-determined behaviors for their own health management, they can successfully maintain self-management as well, which will also have a positive effect on their HRQOL.

Additionally, kidney transplant recipients may experience post-traumatic stress during the transplantation process [13]. Traumatic events make those who have survived or learned from the incident flushed, panic-stricken, and anxious [14]. Therefore, post-traumatic disorders may link non-adherence to medication and poor outcomes in kidney transplant recipients [13]. However, even though trauma may be a negative event, wisdom may be gained through positive post-traumatic experiences and self-reflection [15]. Therefore, if kidney transplant patients achieve post-traumatic growth, such as an optimistic attitude and personal growth during the transplantation process [16, 17], it can be expected to have a positive effect on the transplant outcome and HRQOL [13].

To date, previous research on factors associated with the HRQOL of kidney transplant recipients has focused on physical factors such as perceived health status, symptom experience, and social support. In contrast, little research has addressed the effects of autonomous factors, such as self-determination and post-traumatic growth. Since kidney transplant recipients need to take immunosuppressants and manage their long-term health, it is necessary to examine the associations among autonomous factors on their HRQOL. Therefore, we investigated the associations among perceived health status, social support, self-determination, post-traumatic growth, and HRQOL in kidney transplant recipients to suggest ways to improve kidney transplant recipients’ HRQOL.

### Study objectives

Our specific objectives were as follows: first, to describe the levels of perceived health status, social support, self-determination, post-traumatic growth, and HRQOL in kidney transplant recipients; second, to investigate the differences in HRQOL according to general and transplant-related characteristics of kidney transplant recipients; thirdly, to identify the relationship among perceived health status, social support, self-determination, post-traumatic growth, and HRQOL in kidney transplant recipients; and, fourthly, to identify the factors, including perceived health status, social support, self-determination, and post-traumatic growth, influencing HRQOL of kidney transplant recipients.

## Materials and methods

### Research design

This study used a cross-sectional, self-report survey to investigate the association among perceived health status, social support, self-determination, post-traumatic growth, and HRQOL in kidney transplant recipients.

### Participants

Participants were those who visited the outpatient solid organ transplant center at S University Hospital from 19 December 2019 to 21 January 2020 after receiving kidney transplantation. Inclusion criteria were adults aged ≥ 18 years who understood the purpose of the study, agreed to participate, and could understand and respond to the questionnaire.

G*Power 3.1 [18] was used to estimate the required sample size for multiple regression analyses under the assumption of a power of .95, significance level of .05, and medium effect size of .15. The minimum required sample size was 146. Since there were no existing studies that used regression analyses using the current independent variables, we used a medium effect size as proposed by Cohen [19]. Considering a 20% dropout rate, the survey was distributed to 170 kidney transplant recipients. Seven participants were unable to complete the questionnaire, because they were busy or they thought the questionnaire was too complicated. Thus, data from 163 participants were included in the final analysis.

### Measures

#### General and transplant character

We measured participants’ general characteristics based on sex, age, marital status, education level, religion, income source, monthly income, pre-transplant occupation, and post-transplant occupation. Post-transplant occupations were examined whether or not participants had a job at the time of survey. Income source, monthly income, pre-transplant occupation, and post-transplant occupation were investigated to identify the economic and employment statuses.

We measured participants’ transplant characteristics based on the period since transplant, number of transplants, experience of dialysis before transplant, transplant decision, regret associated with transplant, willingness to recommend transplant, transplant type, and type of living donation; type of living donation was classified as blood relation, spouse, and non-blood relation including relatives and non-relatives.

#### Perceived health status

We measured participants’ subjective assessment of their own health using an instrument developed by Speake, Cowart, and Pellet [20] and modified by Son [21]. The tool was approved by the original authors for use. It is a two-item questionnaire measured with a five-point Likert scale (from 1–5). The questions were as follows: “What is your overall health status now?” and “What is your health status compared to people of similar ages?” A higher score indicated a higher level of perceived health status. Cronbach’s alphas were .74 for Son’s study [21] and .84 for this study.

#### Post-traumatic growth

Post-traumatic growth was measured using the Korean version developed originally by Tedeschi and Calhoun [22], which was translated, modified, and verified for reliability and validity by Song [23]. The tool comprises 16 items that are measured on a six-point Likert scale (from 0–5). Higher scores indicate more positive post-traumatic changes. The tool was approved by the original authors for use. Cronbach’s alphas were .90 for Tedeschi and Calhoun’s study [22], .91 for Song’s study [23], and .91 for this study.

#### Social support

We used the Multidimensional Scale of Perceived Social Support developed by Zimet et al. [24], which was translated by Kim [25]. The tool was approved by the original authors for use. This scale comprises 10 questions measured on a seven-point Likert scale (from 1–7). Higher scores indicate higher social support. Cronbach’s alphas were .91 for Zimet et al.’s study [24], .87 for Kim ‘s study [25], and .93 for this study.

#### Self-determination

Self-determination was measured using the Korean version of the basic psychological needs index developed originally by Ryan and Deci [26], which was translated, modified, and verified for reliability and validity by Lee and Kim [25]. The tool was approved by the original authors for use. This scale comprises 18 questions measured on a five-point Likert scale (from 1–5). Higher scores indicate higher self-determination. Cronbach’s alphas were .87 for Lee and Kim’s study [27], and .86 for this study.

#### HRQOL

HRQOL was measured using the Medical Outcomes Study Short Form 36-item Health Survey (SF-36) [28], which was translated, modified, and verified for reliability and validity by Nam and Lee [29]. The SF-36 can be divided as follows: the physical component summary refers to “physical function”, “role-physical”, “bodily pain”, and “general health” and the mental component summary refers to “vitality”, “social functioning”, “role-emotional”, and “mental health”. The tool was approved by Nam and Lee [29] for use. Cronbach’s alphas were .70 for Nam and Lee’s study [27], and .90 for this study.

### Data collection method

Data were collected by sampling convenience from 163 kidney transplant recipients who visited the outpatient solid organ transplant center of S University Hospital from December 19, 2019 to January 21, 2020. The researchers explained the purpose of the study to participants, who were asked to complete the questionnaire after agreeing to participate. Participants responded to questions about general characteristics, transplant characteristics, perceived health status, post-traumatic growth, social support, self-determination, and HRQOL. Participants were given a small gift worth two dollars as a token of gratitude for their participation.

### Ethical considerations

This study received approval from the Institutional Review Board of Seoul National University Hospital (no. 1911-187-1083). Prior to data collection, a researcher explained the purpose the study, the time to be spent filling out the questionnaire, the benefits and risks of study participation, personal information protection, and that participants could discontinue participation at any time. The study was conducted after receiving written consent of the participants.

### Data analysis method

The data were statistically analyzed using the software SPSS version 25.0 (IBM Corp., Armonk, NY, USA). Participants’ general and transplant characteristics, perceived health status, post-traumatic growth, social support, self-determination, and HRQOL were measured using descriptive statistics (frequency, percentage, mean, standard deviation). The differences in HRQOL according to their general and transplant characteristics were analyzed using independent *t*-tests, one-way analyses of variance, and Kruskal-Wallis tests. Scheffe’s tests were used for post-hoc analyses. The correlations between variables were examined using Pearson’s correlations coefficient. Multiple regression analyses were performed to identify the influence of general and transplant characteristics, perceived health status, post-traumatic growth, self-determination, and social support on HRQOL. Categoric variables were analyzed by converting them into dummy variables. In order to explore the best factors, considering the relationship between the explanatory variables, the process of selecting variables according to the stepwise method was added for items that showed significance in the univariate analysis.

## Results

### Participants’ general characteristics and differences

Participants’ mean age was 55.88 ± 11.72 years. Of the total, 57.7% were male, 72.8% were married, and 59.5% were religious. Regarding education level, more than half (60.5%) were college graduates. The majority of participants (65.0%) had income sources that were their own. Of the total participants, 29.1% had a monthly income greater than $3,400. Meanwhile, 47.2% of participants were company employees in their pre-transplant occupations, and 32.5% were company employees in their post-transplant occupations.

Regarding differences in HRQOL according to participants’ general characteristics, there were significant differences based on education level (F = 5.22, *p =* .024), income source (F = 4.38, *p =* .038), monthly income (F = 12.38, *p* = .001), and post-transplant occupation (F = 5.18, *p =* .024). The post hoc test showed that the mean HRQOL score of participants earning less than $1,700 a month was significantly lower than that of participants earning more than $1,700 a month.

In the subdomains of HRQOL, regarding differences in physical component summary according to participants’ general characteristics, there were significant differences based on age (F = 5.84, *p =* .017), education level (F = 5.22, *p =* .024), income source (F = 4.99, *p =* .027), monthly income (F = 10.03., *p* = .002), and post-transplant occupation (F = 10.35, *p* = .002).

In the subdomains of HRQOL, regarding differences in mental component summary according to participants’ general characteristics, there were significant differences based on marital status (t = -2.03, *p =* .046), education level (F = 4.10, *p =* .045), and monthly income (F = 11.66, *p* = .001). The post hoc test revealed that the mean HRQOL score of mental component summary of participants earning less than $1,700 a month was significantly lower than that of participants earning more than $1,700 a month [Table 1].

**Table 1:** Health-related quality of life according to participants’ general characteristics (*N* = 163).

Variable	Categories	*n* (%)	HRQOL (PCS+MCS)		Physical component summary		Mental component summary	
			M ± SD	t/F *(p)*	M ± SD	t/F *(p)*	M ± SD	t/F *(p)*
Sex	Female	69 (42.3)	73.42 ± 13.01	-1.03 (.303)	72.02 ± 14.00	-1.40 (.164)	74.94 ± 14.28	-.56 (.577)
	Male	94 (57.7)	75.57 ± 13.31		75.09 ± 13.69		76.20 ± 14.21	
Age	≤ 49	47 (28.8)	76.71 ± 11.27	2.73 (.100)	77.04 ± 11.25	5.84 (.017)	76.49 ± 13.30	.53 (.468)
	50–59	43 (26.4)	75.62 ± 12.99		74.96 ± 12.81		76.44 ± 14.57	
	≥ 60	73 (44.8)	72.78 ± 14.31		71.01 ± 15.51		74.67 ± 14.69	
Marital status	Single/other	44 (27.2)	71.62 ± 13.41	-1.70 (.094)	71.75 ± 14.19	-1.05 (.298)	71.69 ± 15.13	-2.03 (.046)
	Married	118 (72.8)	75.61 ± 12.89		74.35 ± 13.61		76.97 ± 13.57	
Education level	≤ Middle school	17 (10.5)	70.23 ± 18.27	5.22 (.024)	68.27 ± 20.44	5.22 (.024)	72.19 ± 17.44	4.10 (.045)
	High school	47 (29.0)	72.61 ± 12.61		72.21 ± 12.49		73.17 ± 14.46	
	≥ College	98 (60.5)	76.60 ± 12.15		75.66 ± 12.87		77.67 ± 13.22	
Religious	Yes	97 (59.5)	73.53 ± 12.86	-1.32 (.188)	72.97 ± 13.41	-.90 (.372)	74.21 ± 13.90	-1.58 (.116)
	No	66 (40.5)	76.33 ± 13.59		74.99 ± 14.53		77.81 ± 14.50	
Income source	Self	106 (65.0)	75.97 ± 13.32	4.38 (.038)	75.31 ± 13.58	4.99 (.027)	76.75 ± 14.54	3.01 (.085)
	Spouse	32 (19.6)	74.03 ± 12.29		72.82 ± 12.86		75.46 ± 13.84	
	Other	25 (15.3)	69.94 ± 13.11		68.58 ± 15.42		71.31 ± 12.87	
Monthly income (dollar)	< 1,700^a^	37 (23.4)	69.88 ± 13.30	12.38 (.001)	69.56 ± 13.96	10.03 (.002)	70.21 ± 14.33^a^	11.66 (.001)
	1,701–2,500^b^	37 (23.4)	72.05 ± 13.86	a<b, c, d	70.34 ± 15.47		73.99 ± 14.32^b^	a < b, c, d
	2,501–3,400^c^	38 (24.1)	77.72 ± 11.03		77.08 ± 11.31		78.69 ± 12.62^c^	
	> 3,400^d^	46 (29.1)	78.54 ± 12.58		77.41 ± 13.09		79.67 ± 13.43^d^	
Pre-transplant occupation	Company employee	77 (47.2)	75.68 ± 12.96	.66 (.418)	75.18 ± 13.45	.63 (.430)	76.25 ± 13.78	.51 (.475)
	Self-employed	34 (20.9)	73.92 ± 14.25		72.01 ± 15.24		76.07 ± 14.63	
	None	7 (4.3)	74.06 ± 11.50		76.27 ± 10.94		71.86 ± 13.84	
	Housewife	32 (19.6)	73.53 ± 14.12		71.20 ± 15.64		76.08 ± 14.86	
	Other	13 (8.0)	73.70 ± 11.48		75.27 ± 8.71		72.13 ± 15.63	
Post-transplant occupation	Company employee	53 (32.5)	78.25 ± 11.60	5.18 (.024)	78.90 ± 11.41	10.35 (.002)	77.60 ± 13.11	1.08 (.299)
	Self-employed	34 (20.9)	75.73 ± 12.85		74.57 ± 12.90		77.15 ± 14.33	
	None	26 (16.0)	70.40 ± 14.60		69.76 ± 14.78		71.05 ± 15.89	
	Homemaker	40 (24.5)	71.01 ± 13.74		68.32 ± 15.26		73.88 ± 14.45	
	Other	10 (6.1)	77.70 ± 11.67		76.42 ± 11.82		79.50 ± 12.72	

HRQOL = Health-related quality of life; PCS = Physical component summary; MCS = Mental component summary

a = less than $1,700

b = more than $1,701 and less than $2,500

c = more than $2,501 and less than $3,400

d = more than $3,400.

### Participants’ transplant characteristics and differences

Of the participants, the post-transplant period had been under five years for 33.7% and over 21 years for 11.0%. This was the first transplant for the majority of participants (93.8%), and most had dialysis before the transplant (76.9%). More than half of the participants (66.7%) made the transplant decision voluntarily, and 97.5% did not regret the transplant decision. Almost all participants (98.1%) were willing to recommend transplants to others. More than half of the participants (61.5%) had living transplants, and more than half of the living-transplants (64.8%) were blood-related transplants.

Regarding differences in HRQOL according to participants’ transplant characteristics, there was no significant difference found.

In the subdomains of HRQOL, regarding differences in physical component summary according to participants’ transplant characteristics, there was a significant difference based on the type of living donation (F = 6.92, *p =* .010). Regarding differences in mental component summary according to participants’ transplant characteristics, there was no significant difference found [Table 2].

**Table 2:** Health-related quality of life according to participants’ transplant characteristics (*N* = 163).

Variable	Categories	*n* (%)	HRQOL (PCS+MCS)		Physical component summary		Mental component summary	
			M ± SD	t/F *(p)*	M ± SD	t/F *(p)*	M ± SD	t/F *(p)*
Period since transplant (year)	1–5	55 (33.7)	73.32 ± 13.83	1.71 (.679)	72.49 ± 14.28	.24 (.623)	74.14 ± 15.12	.14 (.705)
	6–10	55 (33.7)	76.28 ± 13.25		75.03 ± 14.95		77.65 ± 13.37	
	11–20	35 (21.5)	73.97 ± 11.86		73.52 ± 11.93		74.65 ± 13.44	
	≥ 21	18 (11.0)	75.21 ± 13.98		74.48 ± 13.37		76.22 ± 15.70	
Number of transplants	1	151 (93.8)	74.38 ± 13.39	-1.11 (.292)	73.57 ± 14.11	-.73 (.482)	75.27 ± 14.33	-1.41 (.188)
	≥ 2	10 (6.2)	78.43 ± 11.04		76.30 ± 11.27		81.40 ± 13.26	
Dialysis before transplant	No	37 (23.1)	76.04 ± 11.91	.69 (.493)	76.20 ± 12.41	1.18 (.242)	76.11 ± 13.44	.18 (.855)
	Yes	123 (76.9)	74.45 ± 13.50		73.36 ± 14.08		75.64 ± 14.52	
Transplant decision	Voluntary	108 (66.7)	74.36 ± 13.01	-.32 (.751)	73.57 ± 13.60	-.24 (.811)	75.15 ± 14.02	-.46 (.644)
	Recommended	54 (33.3)	75.07 ± 13.69		74.14 ± 14.61		76.25 ± 14.44	
Regret with transplant	No	159 (97.5)	74.83 ± 12.89	.55 (.619)	73.96 ± 13.37	.47 (.671)	75.82 ± 14.10	.66 (.558)
	Yes	4 (2.5)	68.11 ± 24.24		66.91 ± 29.98		69.31 ± 19.75	
Willingness to recommend transplant	No	3 (1.9)	61.47 ± 24.30	-.96 (.437)	61.11 ± 25.52	-.88 (.471)	61.83 ± 23.67	-1.03 (.409)
	Yes	159 (98.1)	74.99 ± 12.90		74.11 ± 13.59		76.01 ± 13.97	
Transplant type	Deceased donor	62 (38.5)	73.27 ± 14.09	-.89 (.374)	72.02 ± 14.93	-1.15 (.252)	74.67 ± 14.84	-.53 (.598)
	Living donor	99 (61.5)	75.23 ± 12.54		74.68 ± 13.14		75.90 ± 13.72	
Type of donation	Blood relation	59 (64.8)	75.93 ± 11.68	3.66 (.059)	75.89 ± 11.66	6.92 (.010)	76.09 ± 13.62	1.03 (.314)
	Spouse	27 (29.7)	72.12 ± 14.29		70.39 ± 14.81		74.04 ± 15.64	
	Non-blood relation	5 (5.5)	66.81 ± 9.52		63.48 ± 10.68		70.15 ± 8.74	

HRQOL = Health-related quality of life; PCS = Physical component summary; MCS = Mental component summary.

### Levels of perceived health status, post-traumatic growth, social support, self-determination, and HRQOL

The mean score for perceived health status was 3.44 ± 0.81, and the mean score for post-traumatic growth was 3.49 ± 0.72. The mean score for social support was 5.48 ± 1.01, and the mean score for self-determination was 3.72 ± 0.48. The overall mean score for HRQOL was 74.66 ±13.19. Among HRQOL of the physical component summary, the highest score was reported for physical function. Among HRQOL of the mental component summary, the highest score was reported for social functioning [Table 3].

**Table 3:** Levels of perceived health status, post-traumatic growth, social support, self-determination, and health related quality of life (N = 163).

Variable	M ± SD	Range	Min	Max	Cronbach’s α
Perceived health status	3.44 ± 0.81	1–5	1.00	5.00	.84
Post-traumatic growth	3.49 ± 0.72	0–5	0.69	5.00	.91
Social support	5.48 ± 1.01	1–7	1.83	7.00	.93
Self-determination	3.72 ± 0.48	1–5	2.44	4.78	.86
Health-related quality of life	74.66 ± 13.19	0–100	35.43	99.50	.90
Physical component summary	73.79 ± 13.87	0–100	23.61	99.00	.79
Physical function	86.15 ± 12.53	0–100	44.44	100.00	.86
Role-physical	76.91 ± 19.40	0–100	20.00	100.00	.92
Bodily pain	73.58 ± 22.82	0–100	10.00	100.00	.86
General health	58.61 ± 15.28	0–100	20.00	96.00	.84
Mental component summary	75.66 ± 14.21	0–100	36.25	100.00	.86
Vitality	65.03 ± 15.55	0–100	25.00	100.00	.72
Social functioning	80.86 ± 18.00	0–100	30.00	100.00	.64
Role-emotional	80.77 ± 18.84	0–100	20.00	100.00	.95
Mental health	74.84 ± 14.73	0–100	28.00	100.00	.77

### Correlation between HRQOL and perceived health status, post-traumatic growth, social support, and self-determination

HRQOL showed statistically significant positive correlation with perceived health status (r = .56, *p* < .001), post-traumatic growth (r = .18, *p* = .022), social support (r = .25, *p* = .002), and self-determination (r = .36, *p* < .001). HRQOL of the physical component summary was positively correlated with perceived health status (r = .53, *p* < .001), social support (r = .16, *p* = .040), and self-determination (r = .25, *p* = .001). Meanwhile, HRQOL of the mental component summary was positively correlated with perceived health status (r = .52, *p* < .001), post-traumatic growth (r = .20, *p* = .012), social support (r = .30, *p* < .001), and self-determination (r = .42, *p* < .001).

Perceived health status showed statistically significant positive correlation with post-traumatic growth (r = .32, *p* < .001, social support (r = .30, *p* < .001), and self-determination (r = .32, *p* < .001). Post-traumatic growth was positively correlated with social support (r = .57, *p* < .001) and self-determination (r = .33, *p* < .001). Social support was positively correlated with self-determination (r = .57, *p* < .001) [Table 4].

**Table 4:** Correlation between health-related quality of life and perceived health status, post-traumatic growth, social support, and self-determination (N = 163).

Variables	Perceived health status	Post-traumatic growth	Social support	Self-determination	HRQOL	PCS	MCS
Perceived health status	1						
Post-traumatic growth	.32 (< .001)	1					
Social support	.30 (< .001)	.57 (< .001)	1				
Self-determination	.32 (< .001)	.33 (< .001)	.57 (< .001)	1			
HRQOL	.56 (< .001)	.18 (.022)	.25 (.002)	.36 (< .001)	1		
PCS	.53 (< .001)	.14 (.071)	.16 (.040)	.25 (.001)	.94 (< .001)	1	
MCS	.52 (< .001)	.20 (.012)	.30 (< .001)	.42 (< .001)	.94 (< .001)	.78 (< .001)	1

HRQOL = Health-related quality of life; PCS = Physical component summary; MCS = Mental component summary.

### Factors affecting participants’ HRQOL

The outcome of the stepwise regression analysis was as follows.

The most powerful factor affecting HRQOL was perceived health status (*β* = 0.47, *p* < .001). The next most powerful factors affecting HRQOL were post-transplant occupation (*β* = 0.17, *p* = .009), followed by income source (*β* = -0.13, *p* = .046). The explanatory power of these variables was 34.8% (F = 28.81, *p* < 0.001, R^2^ = 0.360, Adj. R^2^ = 0.348). The variance inflation factor (VIF) values were all less than 10, with no multicollinearity, and the Durbin-Watson value was sufficient to satisfy independence of residuals [Table 5].

**Table 5:** Factors affecting health-related quality of life (N = 163).

Variable	Categories	B	SE	*Β*	t *(p)*
Income source	Self/Spouse				
	Other	-4.86	2.41	-0.13	-2.01 (.046)
Post-transplant occupation	No				
	Yes (including housewife)	4.61	2.41	0.17	2.65 (.009)
Perceived health status		7.74	1.07	0.47	7.22 (< .001)
R^2^ (%)		36.0			
Adjusted R^2^ (%)		34.8			
F		28.81 (< .001)			
Variance inflation factor		1.02–1.10			
Durbin-Watson		1.89			

In the subdomains of HRQOL, the factors influencing HRQOL of the physical component summary were perceived health status (*β* = 0.55, *p*< .001), age (*β* = -0.35, *p* < .001), and education level (*β* = 0.21, *p* = .019). The explanatory power of these variables was 37.5% (F = 17.51, *p* < 0.001, R^2^ = 0.397, Adj. R^2^ = 0.375). The factors influencing HRQOL of the mental component summary were perceived health status (*β* = 0.45, *p* < .001), self-determination (*β* = 0.27, *p* < .001), and education level (*β* = 0.18, *p* = .006). The explanatory power of these variables was 38.0% (F = 32.24, *p* < 0.001, R^2^ = 0.392, Adj. R^2^ = 0.380) [Table 6].

**Table 6:** Factors influencing physical component summary and mental component summary.

Dependent variable	Predictor	B	*Β*	t *(p)*	VIF	R^2^ (%)	Adjust R^2^ (%)	F*(p)*	D-W
Physical component summary	Age	-0.37	-0.35	-3.89 (< .001)	1.04	39.7	37.5	17.51 (< .001)	1.88
	Education (college or higher)	5.69	0.21	2.39 (.019)	1.06				
	Perceived health status	9.86	0.55	6.27 (< .001)	1.03				
Mental component summary	Education (college or higher)	5.12	0.18	2.80 (.006)	1.13	39.2	38.0	32.24 (< .001)	1.85
	Perceived health status	7.78	0.45	6.67 (< .001)	1.13				
	Self-determination	7.95	0.27	4.00 (< .001)	1.01				

VIF = Variance inflation factor; D-W = Durbin-Watson.

## Discussion

Identifying associations between HRQOL and factors associated with individual autonomy can provide ways to improve HRQOL among kidney transplant recipients. Thus, we conducted this study to identify whether HRQOL in transplant recipients was related to perceived health status, self-determination, post-traumatic growth, and social support.

In this study, perceived health status was the most powerful factor influencing participant HRQOL, corroborating previous data [4]. We measured perceived health status, a subjective assessment of an individual’s health status [20], which predicts HRQOL of kidney transplant recipients better than an objective evaluation [4, 30]. We also found that, of the eight components of SF-36, physical function yielded the highest mean score and general health the lowest, similar to results from Mouelhi et al. [31]. This outcome indicates that kidney transplant recipients may feel unhealthy in general, even if health problems did not limit their physical activity. Hence, health care workers should pay attention to perceived health status as well as objective health conditions to improve HRQOL of kidney transplant recipients.

Self-determination affected the mental component summary of HRQOL, supporting previous reports [32, 33] and providing evidence for self-determination theory, which posits that psychological well-being is associated with three basic psychological needs: autonomy, competence, and relatedness [34]. Our results highlight that an individual, whose basic needs are met, is likely to perform intrinsically motivated behaviors [33, 34], which indicates the need to create an environment that promotes basic needs. Possible strategies include understanding patient emotions, allowing patients freedom to make decisions about desirable behavior (e.g., self-management), and fostering a reliable, supportive relationship between patients and healthcare professionals [33, 34]. Clearly, more data are needed to understand how to develop strategies to improve basic psychological needs of kidney transplant recipients. Additionally, this study found that higher self-determination was strongly associated with higher HRQOL, which is in line with previous research [32]. Thus, patients who engage in self-management and possess elevated self-determination will have an advantage in maintaining good health status after transplantation [11], eventually leading to higher HRQOL. Our work supports this link, as self-determination was associated with perceived health status. However, these results cannot address the issue of causality among the variables of self-determination, self-management, and HRQOL. Further research should clarify the relationships between these three factors.

Our results suggest that post-traumatic growth is positively correlated with HRQOL, is similar to the previous study conducted on cancer survivors [35]. Post-traumatic growth is related to perceived positive changes following traumatic life events rather than objective status [36]. Therefore, the results indicate that kidney transplant recipients who have experienced post-traumatic growth may have evaluated HRQOL more positively, just as cancer survivors who have experienced post-traumatic growth rate HRQOL [35]. As the process of post-traumatic growth may involve small and slow alterations over time in kidney transplant recipients [37], developing a program that improves post-traumatic growth can be difficult. Various programs, including targeted social support, clinical intervention, education, and applying coping strategies such as resilience [38, 39], that are capable of helping kidney transplant recipients attain post-traumatic growth can be used as interventions to raise HRQOL.

Higher social support was associated with higher HRQOL, which is consistent with previous studies [4, 31, 40]. Social support enhances psychological adaptation for kidney transplant recipients and mediates health problems or crisis situations [4]. For example, we showed that married participants had a high HRQOL score for the mental component summary, indicating that spousal support had a positive effect. Beyond spouses, social support from others, such as healthcare workers and peer groups, have a positive effect on HRQOL [4]. Future research should examine the benefits of improving social support among various groups.

Post-transplant employment is an additional related factor that influences participant HRQOL, coinciding with the findings of a previous study [41, 42]. Specifically, our results demonstrated that having a post-transplant occupation resulted in a difference in HRQOL of the physical component summary, depending on whether participants were employed or not and that variation in monthly income led to a difference in mean HRQOL scores for both the physical and mental component summaries, which corroborates results of previous studies [5, 31, 41, 43]. Post-transplant treatment, such as regular outpatient visits, purchase of immunosuppressants, and hospitalizations, impose economic burdens on kidney transplant recipients [8, 44]. Unresolved economic burdens increase the difficulty of maintaining HRQOL. Post-transplant employment implies good health status that allows a return to work and a stable income [43]. This study highlights the need for helping kidney transplant recipients retain employment or find new jobs after transplantation as an important method for improving HRQOL [45, 46]. Therefore, we recommend the implementation of an institutional system that supports transplant recipients returning to work. Moreover, income source influenced HRQOL; specifically, we identified a difference in HRQOL of the physical component summary depending on whether the income source is from the transplant recipient. This outcome is related to the results on post-transplant employment, demonstrating that kidney transplant recipients with sufficient physical health can be the source of income for themselves [43], which improves HRQOL.

The physical and mental component summaries of education level led to a difference in mean HRQOL scores, aligning with earlier work [47]. Korean education levels are closely related to satisfaction in areas of life, including jobs, family relationships, and leisure [48]. Thus, we can predict that HRQOL will track educational level. However, some studies did not find such a correlation [1, 5], suggesting the need for further research to explore this relationship.

Finally, we find that age was the primary factor significantly affecting the physical component summary of HRQOL, which corresponds with earlier work [31]. Our results demonstrate that more effort should be made to improve HRQOL among elderly transplant patients, especially as we observe a gradual increase in the average age of individuals waiting for kidney transplants [3].

## Conclusions and recommendations

This study was conducted to identify the factors—including autonomous factors such as self-determination and post-traumatic growth—associated with HRQOL in kidney transplant recipients. It was found that HRQOL is associated with perceived health status, social support, post-traumatic growth, and self-determination. In particular, self-determination affected HRQOL of the mental component summary. Therefore, the creation of environments in which kidney transplant recipients can act with self-determination and developing intervention programs that can enhance self-determination will contribute to enhancing HRQOL of kidney transplant recipients. As the factors influencing kidney transplant recipients’ HRQOL were perceived health status, post-transplant occupation, and income source, an institutional system to help kidney transplant recipients return to work needs to be developed. The study first revealed that the higher post-traumatic growth and self-determination are, the higher HRQOL in kidney transplant recipients is. The study also showed that self-determination directly affects the mental component of HRQOL. The result highlights that to improve HRQOL for kidney transplant recipients, not only physical and socioeconomic factors, but also autonomous factors such as post-traumatic growth or self-determination should be considered. This study suggested that efforts to improve post-traumatic growth and self-determinism of kidney transplant recipients will contribute to improving HRQOL.

The study has a few limitations. First, the sample was a convenience sample of kidney transplant recipients from a hospital, thus the generalization of the study’s findings is difficult. Consequently, a future study should be conducted using a representative sample to investigate the factors associated with HRQOL. Second, the path could not be analyzed in terms of the association between self-determination and HRQOL. However, identifying mediating variables such as self-management will help to establish this association. Nevertheless, the study is significant in that it successfully identified the associations among self-determination, post-traumatic growth and HRQOL in kidney transplant recipients, and the results have the potential to be useful in exploring effective ways to improve HRQOL.
